# A biomechanics-based parametrized cardiac end-diastolic pressure–volume relationship for accurate patient-specific calibration and estimation

**DOI:** 10.1038/s41598-023-38196-5

**Published:** 2023-07-11

**Authors:** Dominique Chapelle, Arthur Le Gall

**Affiliations:** 1 Inria, Palaiseau, France; 2 LMS, Ecole Polytechnique, CNRS, Institut Polytechnique de Paris, Palaiseau, France; 3 AP-HP, Hôpital Lariboisière, Paris, France

**Keywords:** Biophysics, Cardiology, Biomedical engineering

## Abstract

A simple power law has been proposed in the pioneering work of Klotz et al. (Am J Physiol Heart Circ Physiol 291(1):H403–H412, 2006) to approximate the end-diastolic pressure–volume relationship of the left cardiac ventricle, with limited inter-individual variability provided the volume is adequately normalized. Nevertheless, we use here a biomechanical model to investigate the sources of the remaining data dispersion observed in the normalized space, and we show that variations of the parameters of the biomechanical model realistically account for a substantial part of this dispersion. We therefore propose an alternative law based on the biomechanical model that embeds some intrinsic physical parameters, which directly enables personalization capabilities, and paves the way for related estimation approaches.

## Introduction

The passive behavior of the heart translates into the so-called end-diastolic pressure–volume relationship (EDPVR), which gives the volume of the left ventricle (LV) reached under a given filling pressure. The patient-specific determination of this curve is an important objective, both for clinical—e.g. for diagnosing pathological values of the myocardium compliance—and for biomechanical modeling purposes—for calibrating the constitutive parameters of the passive behavior. In the paper^1^ a most interesting property of the EDPVR was established, i.e. that provided the volume is adequately normalized this curve displays limited inter-individual—or even inter-species—variability, and can be approximated in the single form

1 $$\begin{aligned} P = A (V_n)^B, \text { with } V_n = \frac{V-V_\text {ref}}{V_{30}-V_\text {ref}}, \quad A=27.8, \; B=2.76, \end{aligned}$$

with $V_\text {ref}$ and $V_{30}$ being the volumes associated with zero pressure and 30 mmHg, respectively. Throughout this paper we will refer to this as the “Klotz et al. relationship”. This relationship is extremely valuable to estimate the complete EDPVR with very few measured pressure–volume data points, and also to calibrate the coefficients of a biomechanical model based on the full EDPVR curve estimated with this method, rather than with the scarce original data points, see^2–5^ for a few examples. Conversely, the literature is very scarce concerning how a biomechanical model could be used to shed more light on the Klotz et al. relationship, and to provide a critical assessment tool for this relationship, whether it be for its shape itself, or for the dispersion of the data points around the curve. The paper^6^ goes in this direction, by first proposing a modified curve with a non-zero slope at the origin, and concluding that the fitting of a biomechanical model with this modified curve leads to yet another different shape.

In the present work, we show by using the biomechanical model of^7^ that the remaining dispersion of the data points around the Klotz et al. relationship in the normalized volume/pressure space may be accounted for by parametric variations in the model, in particular with a stiffness parameter. Based on this finding, we propose a new parametrized pressure–volume relationship that can be made patient-specific by adjusting the embedded parameters. Therefore, our proposed relationship has the built-in ability to be more accurate—for a given individual—than the “averaged” relationship of Klotz et al.

In “Methods” section (Methods) we summarize the passive cardiac biomechanical model considered in this paper, and perform a calibration of this model based on actual data collected in a clinical setting of general anesthesia. In “Results” section (Results) we report on parameter sensitivity with respect to variations of a stiffness parameter and of the thickness of the ventricular wall. Moreover, we compare personalization capabilities of the biomechanical model and of the Klotz et al. relationship, when considering typical patient-specific configurations associated with different values of the stiffness parameter. We then discuss these results in “Discussion” and “Limitations” sections, and finally provide conclusions in “Conclusions” section.

## Methods

### Biomechanical model

We consider the simplified cardiac model proposed in^7^, itself based on the 3D model proposed and analysed in^8^ and derived by approximating the left ventricular geometry as a hollow sphere, and assuming that fiber directions are isotropically distributed across the thickness in order to have complete spherical symmetry. The constitutive behavior, by contrast, is preserved in its full complexity as in^8^, and since we are concerned here with the EDPVR we focus on the static—or quasi-static—passive part, modeled based on a hyperelastic potential inspired from^9^ and in the following exponential form

2 $$\begin{aligned} W_e = C_0 e^{C_1(J_1-3)^2} + C_2 e^{C_3(J_4-1)^2}, \end{aligned}$$

with $J_1$ and $J_4$ being the so-called first and fourth reduced invariants of the right Cauchy–Green deformation tensor $\textbf{C}$, i.e.,

$$\begin{aligned} J_1 = (\det \textbf{C})^{-\frac{1}{3}} \, \textrm{tr}\,\textbf{C}, \quad J_4= (\det \textbf{C})^{-\frac{1}{3}} \,(1+e_\text {fib})^2,\end{aligned}$$

where $e_\text {fib}$ denotes the relative extension in the fiber direction, and $[C_0,C_1,C_2,C_3]$ is a set of constitutive parameters. We also assume that the cardiac tissue is perfectly incompressible, i.e., $\det \textbf{C}=1$. Under the simplified geometric and kinematical assumptions considered here, the fiber extension is uniform throughout the whole cardiac tissue at any time, and related to the changes in spherical radius by

3 $$\begin{aligned} e_\text {fib}= \frac{R}{R_\text {ref}}-1, \end{aligned}$$

where *R* and $R_\text {ref}$ respectively denote the radius in the current and reference configurations. Note that, although the spherical approximation may seem quite drastic, this simplified model has demonstrated an excellent adequacy—when confronted with actual clinical or experimental data—to represent the pressure–volume behavior of a single ventricle in health and disease^7,10,11^.

Denoting now by $d$ and $d_\text {ref}$ the thickness of the wall in the current and reference configurations, respectively, tissue incompressibility dictates that $4\pi (R_\text {ref})^2d_\text {ref}=4\pi R^2d$, hence,

4 $$\begin{aligned} d= d_\text {ref}(1+e_\text {fib})^{-2}. \end{aligned}$$

The internal volume of the ventricle in the current and reference configurations is given by

5 $$\begin{aligned} V=\frac{4}{3}\pi \Bigl (R-\frac{d}{2}\Bigr )^3, \quad V_\text {ref}= \frac{4}{3}\pi \Bigl (R_\text {ref}-\frac{d_\text {ref}}{2}\Bigr )^3, \end{aligned}$$

and therefore

6 $$\begin{aligned} \frac{V}{V_\text {ref}} = \left( \frac{1+e_\text {fib}-\frac{\epsilon }{2}(1+e_\text {fib})^{-2}}{1-\frac{\epsilon }{2}}\right) ^3, \end{aligned}$$

where $\epsilon$ denotes the dimensionless thickness parameter $\epsilon = d_\text {ref}/R_\text {ref}$.

Finally, in the framework of our biomechanical model, it can be established that the EDPVR can be written in the form

7 $$\begin{aligned} P = P_\text {model}\bigl ( C_i, \epsilon , e_\text {fib}\bigr ), \end{aligned}$$

where $P_\text {model}$ is a function that interrogates the derivatives of the hyperelastic potential (2) with respect to $J_1$ and $J_4$, see^7^ (Eq. (19)), restricted to the static passive case. Note that in order to interpret (7) as a function of the volume *V* we can invert the relationship (6) in the form of an implicit function $e_\text {fib}\bigl (V/V_\text {ref}\bigr )$.

### Model calibration

Our purpose here is to calibrate the above biomechanical cardiac model for a typical human heart. To that aim we use some ultrasound data obtained for a collection of patients anesthetized for a neuroradiological procedure, and select one specific individual with a heart of “average” dimensions within the population considered in the study^12^ in which 45 patients were included, see Table 1 for the specific characteristics of the patient that we selected. We emphasize that this selection of an individual dataset is not performed with a view to a patient-specific study, but to provide a reference EDPVR that can be compared with the data of^1^ and from which whole families of EDPVRs can be generated by varying geometric and mechanical parameters.

#### Data description and determination of (*P*, *V*) point

The ultrasound data were obtained using trans-thoracic echocardiography (TTE), performed after the general anesthesia induction. This study was approved by the appropriate Institutional Review Board – ethical committee of the Société de Réanimation de Langue Française (CE-SRLF 14-34)—which waived the need for written informed consent. Consequently, oral informed consent was obtained after providing a protocol information letter, and the subject had the possibility to withdraw from the study at any time.

The apical 4-chambers view was recorded to estimate the left ventricular (LV) volume at end-diastole $V_\text {ED}$, based on the recommendations given in^13^. In short, the LV cavity is discretized into *N* disks characterized by their heights $(h_i)$ and diameters $(D_i)$. Therefore, $V_\text {ED}$ can be approximated as

8 $$\begin{aligned} V_\text {ED}= \sum _{i=1}^{N} h_i \, \pi \left( \frac{D_i}{2}\right) ^2. \end{aligned}$$

For our selected patient, using $N=550$ disks with $h_i=0.1546$ mm, this rule led to $V_\text {ED}=133$ ml.

The associated end-diastolic pressure $P_\text {ED}$ is then estimated using a semi-quantitative method described in detail in^14^. In short, by analyzing the characteristics of the transmitral flow, a classification of the LV filling pressure as normal or high is determined. In our case, this procedure concluded to a normal LV filling pressure, i.e. $P_\text {ED}=7$ mmHg.

#### Calibration of geometric parameters

The main objective of^1^ is to provide a method for estimating a patient-specific EDPVR based on very few measured pressure–volume points, including with one single such point. This has proven to be extremely valuable to calibrate the passive behavior of biomechanical cardiac models in a number of studies in which the model parameters have been calibrated by using the EDPVR obtained by the method of^1^ applied with the available data, instead of directly calibrating the biomechanical model against the data. This strategy clearly makes the calibration—or estimation—step much better posed than when using the scarce data points. In our case, we only use the method of^1^ to estimate the patient-specific volume parameters $V_\text {ref}$ and $V_{30}$ instead of the full EDPVR. We thus use the estimate of^1^ (Eq. (8)), i.e.

9 $$\begin{aligned} V_\text {ref}= V_\text {ED}(0.6-0.006P_\text {ED}), \end{aligned}$$

which we then substitute in (1) to obtain

10 $$\begin{aligned} V_{30}= V_\text {ref}+ (V_\text {ED}-V_\text {ref})\Bigl (\frac{A}{P_\text {ED}}\Bigr )^\frac{1}{B}. \end{aligned}$$

In addition, we estimate the LV wall thickness in the reference configuration—corresponding to $V_\text {ref}$. To that purpose, we first determine the external volume $V_\text {ext}$ by using the technique already mentioned above to obtain $V_\text {ED}$ in (8) albeit here with the diameters associated with the epicardium. Within the simplified spherical geometry assumption of the biomechanical model considered here—recall “Biomechanical model” section—the thickness $d_\text {ref}$ is directly inferred by subtracting the radii associated with $V_\text {ext}$ and $V_\text {ref}$, i.e.

$$\begin{aligned} d_\text {ref}= \Bigl ( \frac{3(V_\text {ref}+V_\text {ext}-V_\text {ED})}{4\pi } \Bigr )^\frac{1}{3} - \Bigl ( \frac{3V_\text {ref}}{4\pi } \Bigr )^\frac{1}{3}, \end{aligned}$$

where we have used the fact that—due to incompressibility—the total volume of the ventricular wall is the same between the reference and end-diastolic configurations, hence, given by $V_\text {ext}-V_\text {ED}$. Finally, the radius of the midsurface in the spherical geometry is given by

$$\begin{aligned} R_\text {ref}= \Bigl ( \frac{3V_\text {ref}}{4\pi } \Bigr )^\frac{1}{3} + \frac{d_\text {ref}}{2}. \end{aligned}$$

#### Calibration of mechanical parameters

Our objective here is to calibrate the mechanical parameters $(C_i)$ for the selected patient. As already mentioned, one possible strategy would be to use the complete EDPVR provided by the methods of^1^ based on the single point $(P_\text {ED},V_\text {ED})$, in order to formulate an identification problem as well-posed as possible. However, this would introduce a bias towards the type of curve shape associated with the Klotz et al. relationship, and therefore we instead choose to use some of the data considered in^1^, namely, the ex vivo human heart data corresponding to 80 normal and diseased hearts of various etiologies. We will thus propose an identification criterion adapted to the data points provided in^1^ (Fig. 3), i.e. with normalized volumes.

Noting that the normalized volume $V_n$ can be written as

11 $$\begin{aligned} V_n = \frac{V/V_\text {ref}-1}{V_{30}/V_\text {ref}-1}, \end{aligned}$$

we have a very simple linear relationship between $V_n$ and $V/V_\text {ref}$ that only depends on the parameter $V_{30}/V_\text {ref}$, which in our case is calibrated as described above. Therefore, for any choice of the set of mechanical parameters $(C_i)$ we can plot the modeled EDPVR associated with (7) in the $(V_n,P)$ space, and compare it with the data points $(V_n^j,P_\text {data}^j)_{j=1}^{N_\text {data}}$ considered in^1^. In this paper we then calibrate the mechanical parameters by minimizing the following root mean square error (RMSE)

12 $$\begin{aligned} \frac{1}{N_\text {data}}\sqrt{\sum _{j}^{N_\text {data}}{\left[ P_\text {data}^j - P_\text {model}\Bigl (C_i, \epsilon , e_\text {fib}\bigl (V^j/V_\text {ref}\bigr )\Bigr )\right] ^2}}, \end{aligned}$$

where $V^j/V_\text {ref}$ is directly inferred from $V_n^j$ by inverting the linear relationship (11).

In practice, we will carry out a minimization process in two steps. First, using the Klotz et al. relationship, we generate a synthetic set of $P_\text {data}$ to be used in the minimization problem (12) with a steepest gradient descent algorithm. This will provide preliminary mechanical parameters that can subsequently be taken as an initial guess for minimizing (12) with the actual data points of^1^, and using the quasi-Newton algorithm provided in the Matlab fminunc function.

## Results

### Biomechanical model calibration

The geometric parameters calibrated as explained in “Model calibration” section are listed in Table 1.

**Table 1:** Patient’s characteristics and data, and calibrated geometric parameters.

Patient’s characteristics	
Age (years)	51
Gender	Male
Body surface area (m$^2$)	1.6
Prior diseases	None
Treatments	None
Diagnosis	Intracranial aneurism

Patient’s data	
$V_\text {ED}$ (ml)/(ml m$^{-2}$)	133/83
$V_\text {ext}$ (ml)	206

Model parameters	
$V_\text {ref}$ (ml)	74.21
$R_\text {ref}$ (cm)	3.05
$d_\text {ref}$ (cm)	0.88
$\epsilon$	0.2881

The mechanical parameters resulting from this minimization are found to be

$$\begin{aligned}{}[C_0=666\,\text {J.m}^{-3}, C_1=2.9, C_2=104\,\text {J.m}^{-3}, C_3=6.5]. \end{aligned}$$

**Figure 1:**
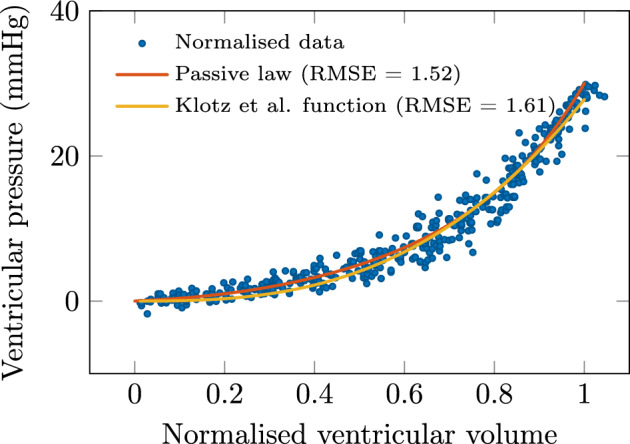
Result of the optimization procedure. Comparison between biomechanical model and Klotz et al. relationship and data in normalized space.

The resulting curve in the $(V_n,P)$ space is shown in Fig. 1 and compared with the relationship and data of Klotz et al.^1^.

### Sensitivity with respect to stiffness parameter

We call “stiffness parameters” the material parameters that have a direct multiplicative effect in the mechanical stress-strain law, i.e. in our case $C_0$ and $C_2$. Therefore we will investigate the impact of multiplicative stiffness variations by considering

$$\begin{aligned}{}[C_0,C_1,C_2,C_3] = [\alpha \bar{C}_0,\bar{C}_1,\alpha \bar{C}_2,\bar{C}_3], \end{aligned}$$

with $[\bar{C}_0,\bar{C}_1,\bar{C}_2,\bar{C}_3]$ being the optimized parameters obtained in “Biomechanical model calibration” section, and $\alpha$ a single coefficient driving stiffness variations. In this case the multiplicative effect of $C_0$ and $C_2$ in the stress-strain law directly translates for the pressure law (7) into

13 $$\begin{aligned} P_\text {model}\Bigl (C_i, \epsilon , e_\text {fib}\bigl (V/V_\text {ref}\bigr )\Bigr ) = \alpha P_\text {model}\Bigl (\bar{C}_i, \epsilon , e_\text {fib}\bigl (V/V_\text {ref}\bigr )\Bigr ). \end{aligned}$$

By contrast, the Klotz et al. relationship does not embed material coefficients per se, recall Eq.  (1). However, stiffness variations will have a direct impact on $V_{30}$ when considering $V_\text {ref}$ fixed. Rewriting (1) in the form

14 $$\begin{aligned} P = P_\text {data}\left( \frac{V_{30}}{V_\text {ref}},\frac{V}{V_\text {ref}}\right) , \end{aligned}$$

a multiplicative change in $V_{30}$ yields

15 $$\begin{aligned} P_\text {data}\left( \frac{\beta V_{30}}{V_\text {ref}},\frac{V}{V_\text {ref}}\right) = \alpha P_\text {data}\left( \frac{V_{30}}{V_\text {ref}},\frac{V}{V_\text {ref}}\right) , \quad \text {with}\; \alpha = \left[ \frac{V_{30}/V_\text {ref}-1}{\beta V_{30}/V_\text {ref}-1} \right] ^B, \end{aligned}$$

which provides a relationship between the change in stiffness—represented by $\alpha$—and the change in $V_{30}$ given by $\beta$.

**Figure 2:**
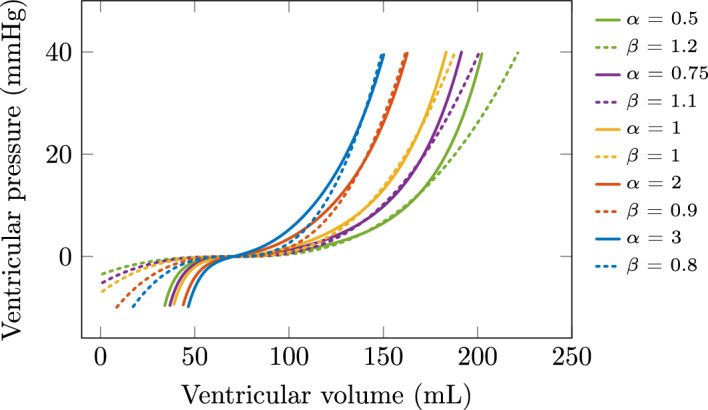
Effect of stiffness variations. Comparison of EDPVR obtained with Klotz et al. relationship (dotted lines) and with biomechanical model (solid lines), when varying the stiffness parameter $\alpha$.

**Figure 3:**
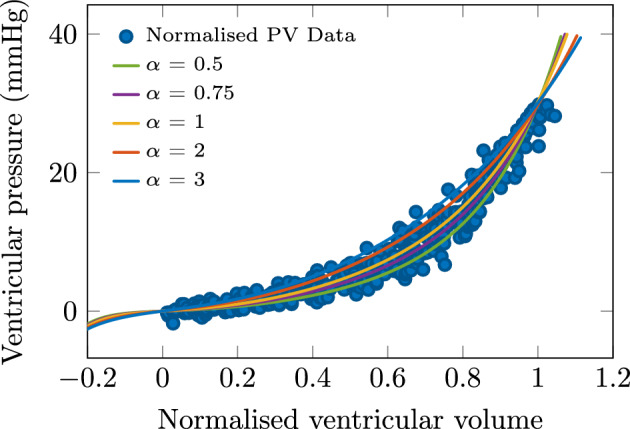
Effect of stiffness variations in biomechanical model, displayed in normalized volume space together with data of^1^.

We compare in Fig. 2 the impact of stiffness changes—associated with the same $\alpha$ coefficient—on the EDPVR curves obtained with the biomechanical model and with the Klotz et al. relationship. In Fig. 3 we show the same effect in the normalized volume space for the biomechanical model only, since the Klotz et al. relationship is unique in this space.

### Sensitivity with respect to thickness parameter

The biomechanical model embeds the thickness parameter $\epsilon$ as an explicit parameter, and it is interesting to study the effect of thickness variations on the EDPVR. By contrast, there is no such parameter in the Klotz et al. relationship, and therefore we will focus on the biomechanical model in this section.

**Figure 4:**
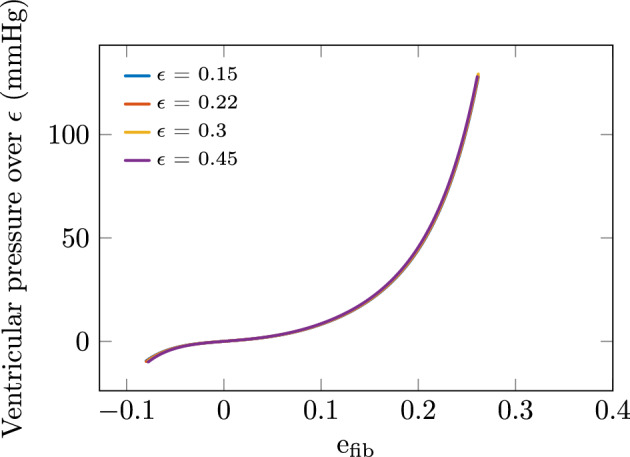
Effect of thickness variations in biomechanical model scaled by thickness parameter, i.e. $P_\text {model}(C_i,\epsilon ,e_\text {fib})/\epsilon$.

As the biomechanical model corresponds to a complex membrane, it is expected—from classical structural mechanics—that the associated stiffness is roughly proportional to the membrane thickness. To investigate this more precisely, we plot in Fig. 4 the scaled quantity $P_\text {model}(C_i,\epsilon ,e_\text {fib})/\epsilon$ as a function of $e_\text {fib}$ for various values of $\epsilon$. Since we observe that the resulting curves are remarkably insensitive to variations in $\epsilon$, we can directly conclude that—in an excellent approximation—the function $P_\text {model}(C_i,\epsilon ,e_\text {fib})$ is linear in $\epsilon$, indeed.

### Comparison of personalization capabilities

In this section we will illustrate and compare the personalization capabilities of the biomechanical model and of the Klotz et al. relationship, in the case of patient-specific stiffness variations.

We start from the calibration performed in “Biomechanical model calibration” section and consider the cases of two different hearts associated with stiffness multiplicative coefficients $\alpha =0.5$ and $\alpha =2$, with all other parameters left unchanged. The biomechanical model is assumed to provide the reference for the resulting modified EDPVR curves, since stiffness variations are adequately represented by construction in such a model via the definition of the stress-strain law.

**Figure 5:**
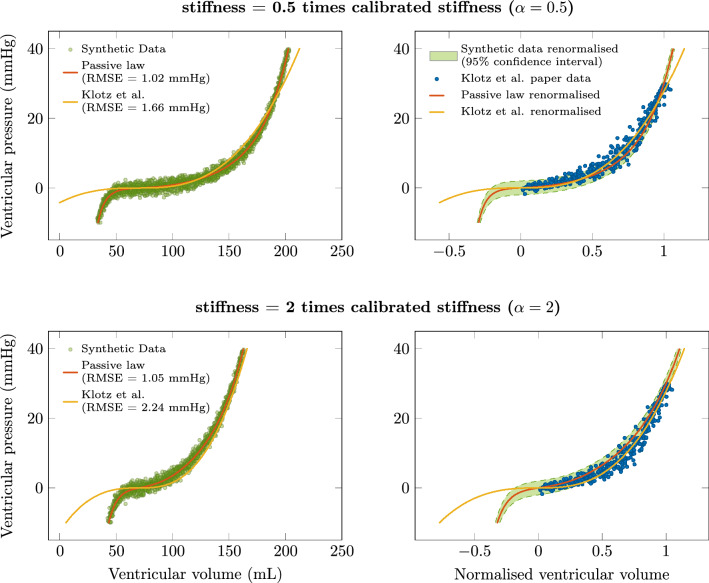
Comparison of personalized models for stiffness coefficients $\alpha =0.5$ and $\alpha =2$. The synthetic data are generated from the biomechanical model with stiffness coefficients $\alpha = [0.5 , 2]$, with random noise added according to a centered reduced normal law with standard deviation 1 mmHg. The RMSE calculated as in (12) between synthetic data and the biomechanical model or the Klotz et al. relationship are provided. The RMSE between the derived EDPVRs are respectively 1.2 mmHg and 2 mmHg for $\alpha = 0.5$ and $\alpha =2$.

We compare in Fig. 5 the EDPVR curves obtained with the biomechanical model and the Klotz et al. relationship, the latter being adapted by applying $V_{30}$ given by the biomechanical model.

## Discussion

We now discuss some specific aspects pertaining to the above results.

*Biomechanical model calibration* The RMSE value obtained with the calibrated biomechanical model is smaller by 6% than that associated with the Klotz et al. relationship (1.52 mmHg versus 1.61 mmHg). From a qualitative point of view, as observed in Fig. 1, the major difference lies in the initial part of the EDPVR where the biomechanical model provides a better fit on average, which was expected due to the vanishing slope of the Klotz et al. relationship at the origin—an unphysical feature because this corresponds to the stiffness near the reference configuration. It should also be pointed out that the optimization process used in this calibration has revealed a rather limited sensitivity of the mechanical parameters—in particular for $C_0$ and $C_2$—albeit this is of little consequence in this study, because we only use the shape of the resulting EDPVR in the normalized $(V_n,P)$ space—which directly corresponds to the optimization criterion—and not the specific values of the mechanical parameters.

*Sensitivity with respect to stiffness and thickness parameters* As directly observed in Fig. 1, there exists some significant dispersion in the EDPVR data considered in the normalized $(V_n,P)$ space. The Klotz et al. relationship—based on a nonlinear regression of the data in this space—treats this dispersion as measurement noise. By contrast, the biomechanical model incorporates some physical parameters, and we see in Fig. 3 that stiffness variations induce deformations of the EDPVR that can instead provide some interpretation for the data dispersion. The same holds with thickness variations since, as seen in “Sensitivity with respect to thickness parameter” section such variations have a similar—linear—effect on the structural stiffness. This is both valuable from a qualitative—physical interpretation—and quantitative point of view, since by adjusting these parameters on a personalised basis we can expect to obtain more accurate results.

*Comparison of personalization capabilities* Upon recalibrating the EDPVR according to stiffness variations associated with multiplicative coefficients $\alpha =0.5$ and $\alpha =2$, the RMSE with the synthetic data is larger by 63% and 113%, respectively, when using the Klotz et al. relationship instead of the biomechanical model, see Fig. 5 (left). All the RMSE values may seem rather small in absolute value, but they should be compared to the noise present in the synthetic data, i.e. 1 mmHg. Moreover, the comparison between the synthetic data range and the actual data points of^1^ in the right-hand side of Fig. 5 further illustrates the fact that the dispersion in the actual data may be realistically accounted for—up to some reasonable amount of measurement noise—by parametric variations associated with the stiffness parameter, or equivalently with the thickness of the ventricular wall. This also provides an interpretation of the benefits provided by the biomechanical model that allows to fit the behavior with the relevant subregion of the Klotz et al. data, as shown by the respective positions of the two different derived EDPVRs in the same graphs. Of course, this personalization study is only preliminary because it is performed based on synthetic data—i.e. EDPVR data generated by the biomechanical model—and a definite validation of this would require actual data of a very invasive type, as also discussed in “Limitations” section below.

*Effective computation of biomechanical model law* In some applications it may be valuable—or even necessary—to be able to compute the function (7) in a very effective manner. This is the case in particular when inverse problems are considered e.g. when seeking to estimate patient-specific parameters based on actual data. To that purpose, we can benefit from the sensitivity explorations performed in “Sensitivity with respect to stiffness parameter” and “Sensitivity with respect to thickness parameter” sections and from the linearity of this function with respect to both the stiffness—when considering a single multiplicative coefficient $\alpha$—and thickness parameters. As a consequence, indeed, we can consider the function

16 $$\begin{aligned} e_\text {fib}\mapsto P_\text {model}\bigl (\bar{C}_i, \bar{\epsilon }, e_\text {fib}\bigr ), \end{aligned}$$

with $\bar{\epsilon }$ being the (fixed) thickness coefficient calibrated for our—supposedly “average”—case above, and perform a fit to an adequate—e.g. polynomial—function to any desired accuracy. As an example, a fit with a polynomial of degree 7 gives an RMSE of 0.03 mmHg for $e_\text {fib}$ between 0 and 0.25 (corresponding to ventricular pressures between 0 and 30 mmHg), which of course is more than sufficient in actual applications. We provide the details of this polynomial fit as supplementary material for reproducibility purposes. Denoting then by $P_\text {fit}(e_\text {fib})$ this approximation of the function (16), for other values of the stiffness and thickness parameters we can approximate $P_\text {model}\bigl (\alpha \bar{C}_i, \epsilon , e_\text {fib}\bigr )$ by $\alpha (\epsilon /\bar{\epsilon })P_\text {fit}(e_\text {fib})$ with excellent accuracy. Moreover, we emphasize that some very valuable physical parameter dependency is retained in this surrogate model, namely, with the linear dependency in $\alpha$ and $\epsilon$, and $V_\text {ref}$ present via $e_\text {fib}(V/V_\text {ref})$.

## Limitations

This study has two main limitations. The first limitation pertains to the scarcity of the data considered. Even though we conjecture that using different datasets for other normal hearts of average dimensions would not significantly impact the results, since—again—we essentially use the fitted *shape* of the biomechanical model in the normalized volume vs. pressure space, this warrants some detailed verification in further studies. An extensive verification would in fact require some invasive data with measured multiple-points EDPVRs for several normal hearts, as well as for several pathological hearts for which the passive stiffness is suspected to be altered.

The second limitation concerns the model used here, in particular due to the spherical approximation made for the geometry in order to avoid the complexity of a 3D model. Other types of reduced models—such as those proposed in^15,16^—could be considered to verify that our findings are not significantly impacted. A 3D computational model could also be employed and calibrated as in^6^ against the data of Klotz et al., albeit it is well-known that 3D models are very sensitive to the specific design of their artificial boundary conditions where the geometric model is truncated, and therefore would not necessarily produce more trustworthy EDPVRs in practice. Other effects could also be included in the model to assess their impact, such as variations in tissue volume. Such variations have been evidenced, indeed—see e.g.^17^—and have often been attributed to the effects of blood perfusion^18^. A reduced model where such effects are embedded—see e.g.^19^—could thus be used for comparative assessment purposes, and the same holds for 3D poromechanical models^20^, although detailed validations of such models are still needed in the cardiac setting.

## Conclusions

We have proposed a law based on a biomechanical model to parametrize a cardiac EDPVR, as an alternative to the phenomenological law proposed in the pioneering work of Klotz et al.^1^. Our proposed law is expressed via the natural variable associated with the fiber extension, and features two major advantages: (1) it incorporates some intrinsic, easily interpretable, physical parameters—namely, the volume in the reference configuration, the wall thickness, and a stiffness parameter—that directly enable personalization capabilities; (2) variations of these parameters may realistically account for the dispersion observed in actual data. An additional valuable finding is that thickness variations have a linear (multiplicative) effect in this law, in an excellent approximation. Finally, polynomial fits can be used as surrogate models of arbitrary accuracy to avoid having to deal with the intricacies of the biomechanical model.

A very natural perspective concerns the use of our proposed law for inverse problem purposes, in order to adapt the EDPVR to measured data points by estimating patient-specific values of the embedded parameters. Using physically interpretable parameters will enrich the output of the estimation process, indeed, and also make it more amenable to quantitative assessments (since such parameters can sometimes be independently measured by specific means).

## Supplementary Information

Supplementary Information.

## Data Availability

The datasets used and/or analysed during the current study are available from the corresponding author on reasonable request.
