# Supplementary material for: A biomechanics-based parametrized cardiac end-diastolic pressure–volume relationship for accurate patient-specific calibration and estimation
Source: Sci Rep. 2023 Jul 11;13:11232. doi: 10.1038/s41598-023-38196-5 (PMC10336140; doi:10.1038/s41598-023-38196-5)
Supplement: Supplementary file 1 — Supplementary Information. [file 41598_2023_38196_MOESM1_ESM.pdf]

Supplementary material: Polynomial fit of degree seven for biomechanical model

The best fit by a polynomial of degree seven for the function $e_{\text{fib}} \mapsto P_{\text{model}}\big(\overline{C}_i, \bar{\epsilon}, e_{\text{fib}}\big)$ is obtained with the polynomial $P_{\text{pol}}(e_{\text{fib}})$ defined as follows

$$P_{\text{pol}}(e_{\text{fib}}) = \sum_{i=0}^{7} a_i\,\phi_i(e_{\text{fib}}), \tag{1}$$

with $a = [86.1176, 1.1892{\cdot}10^5, 8.579{\cdot}10^4, 2.2275{\cdot}10^5, 2.6087{\cdot}10^5, -1.6509{\cdot}10^5, 4.7081{\cdot}10^4, -9.3449{\cdot}10^3]$, and (ϕ_i) a set of shape functions defined as

$$\begin{cases} r = 2\,e_{\text{fib}}/e_{\text{fib}}^{\text{max}} - 1 \\ \phi_0(e_{\text{fib}}) = (1-r)/2 \\ \phi_1(e_{\text{fib}}) = (r+1)/2 \\ \phi_2(e_{\text{fib}}) = (r^2-1)/2 \\ \phi_3(e_{\text{fib}}) = (r^3-1)/6 \\ \phi_4(e_{\text{fib}}) = (r^4-1)/24 \\ \phi_5(e_{\text{fib}}) = (r^5-1)/120 \\ \phi_6(e_{\text{fib}}) = (r^6-1)/720 \\ \phi_7(e_{\text{fib}}) = (r^7-1)/5040 \end{cases} \tag{2}$$

with $e_{\text{fib}}^{\text{max}} = 0.5$.
